# Hormone and receptor activator of NF-κB (RANK) pathway gene expression in plasma and mammographic breast density in postmenopausal women

**DOI:** 10.1186/s13058-022-01522-2

**Published:** 2022-04-14

**Authors:** Rachel Mintz, Mei Wang, Shuai Xu, Graham A. Colditz, Chris Markovic, Adetunji T. Toriola

**Affiliations:** 1 Biomedical Engineering Department, Washington University, St. Louis, MO 63110 USA; 2 Division of Public Health Sciences, Department of Surgery, Washington University School of Medicine, Campus Box 8100, 660 South Euclid Ave, St. Louis, MO 63110 USA; 3 Siteman Cancer Center, Barnes-Jewish Hospital and Washington University School of Medicine, St. Louis, MO USA; 4 McDonnell Genome Institute at Washington University, St. Louis, MO 63018 USA

**Keywords:** Mammographic breast density, Hormones, RANK, RANKL, Gene expression

## Abstract

**Background:**

Hormones impact breast tissue proliferation. Studies investigating the associations of circulating hormone levels with mammographic breast density have reported conflicting results. Due to the limited number of studies, we investigated the associations of hormone gene expression as well as their downstream mediators within the plasma with mammographic breast density in postmenopausal women.

**Methods:**

We recruited postmenopausal women at their annual screening mammogram at Washington University School of Medicine, St. Louis. We used the NanoString nCounter platform to quantify gene expression of hormones (prolactin, progesterone receptor (PGR), estrogen receptor 1 (ESR1), signal transducer and activator of transcription (STAT1 and STAT5), and receptor activator of nuclear factor-kB (RANK) pathway markers (RANK, RANKL, osteoprotegerin, TNFRSF18, and TNFRSF13B) in plasma. We used Volpara to measure volumetric percent density, dense volume, and non-dense volume. Linear regression models, adjusted for confounders, were used to evaluate associations between gene expression (linear fold change) and mammographic breast density.

**Results:**

One unit increase in ESR1, RANK, and TNFRSF18 gene expression was associated with 8% (95% CI 0–15%, *p* value = 0.05), 10% (95% CI 0–20%, *p* value = 0.04) and % (95% CI 0–9%, *p* value = 0.04) higher volumetric percent density, respectively. There were no associations between gene expression of other markers and volumetric percent density. One unit increase in osteoprotegerin and PGR gene expression was associated with 12% (95% CI 4–19%, *p* value = 0.003) and 7% (95% CI 0–13%, *p* value = 0.04) lower non-dense volume, respectively.

**Conclusion:**

These findings provide new insight on the associations of plasma hormonal and RANK pathway gene expression with mammographic breast density in postmenopausal women and require confirmation in other studies.

**Supplementary Information:**

The online version contains supplementary material available at 10.1186/s13058-022-01522-2.

## Background

Mammographic breast density (MBD), a strong risk factor for breast cancer, reflects the amount of epithelial and stromal tissues relative to adipose tissue in the breast [1]. Fat appears darker than epithelium and stroma on a mammogram. Women with greater than 75% density on a mammogram have a 4–6 times greater risk of developing breast cancer compared to women with less than 5% density [2]. The Interventional Breast Cancer Intervention Study demonstrated that a pharmacologically induced 10% decrease in MBD over time is clinically meaningful in the context of breast cancer risk reduction [3].

MBD declines post menopause as endogenous hormone levels decline, indicating an association with hormones and age [4]. MBD also increases with menopausal hormone therapy use [5–8], and stopping hormone therapy conversely reverts MBD to prior levels [9]. Nevertheless, studies investigating the associations of circulating hormone levels with MBD report conflicting results [10–17]. Few studies have addressed the genomic signatures of MBD or investigated how hormone gene expression (e.g., progesterone and prolactin) in tissue or blood may be associated with MBD [18–20]. Gene expression may capture transcriptional changes associated with MBD and could help identify biomarkers of MBD. Breast tissue estrogen receptor 1 (ESR1) gene expression was shown to be negatively associated with percent density in postmenopausal women [21].

We have reported that the receptor activator of nuclear factor kappa-Β ligand (RANKL) gene expression is positively associated with MBD in premenopausal women [22], but there are no data on the associations of plasma RANKL and other tumor necrosis factor receptor superfamily members (e.g., TNFRSF18 and TNFRSF13B) gene expression with MBD in postmenopausal women. Preclinical studies have shown that RANK/RANKL signaling is the major mediator of progesterone-induced mammary epithelial proliferation and expansion of mammary stem cells [23]. Progesterone and prolactin also upregulate RANKL expression [24, 25] and interact with the RANK/RANKL pathway through signal transducer and activator of transcription (STAT) signaling [26, 27]. Despite the extensive crosstalk between the hormones and the RANK pathway identified in preclinical studies, their correlations have not yet been evaluated in population-based studies.

Our objectives in this study are twofold: investigate for the first time the (1) associations of plasma hormone and RANK pathway gene expression with volumetric measures of MBD in postmenopausal women; (2) correlations between hormone and RANK pathway gene expression. Study findings should provide new insight into gene expression profiles that may influence MBD in postmenopausal women.

## Methods

### Study population

We recruited 400 postmenopausal women during annual routine screening mammography at the Joanne Knight Breast Health Center (BHC) at the Siteman Cancer Center at Washington University School of Medicine, St. Louis, MO between October 2017 and September 2018. Complete data on gene expression and MBD were available and analyzed for 368 women.

Women were eligible to participate in the study if they were: (1) aged 50–64 years; (2) postmenopausal; (3) able to comply with all required study procedures and schedule, including the provision of blood samples at the time of enrollment. Exclusion criteria included: (1) cancer history; (2) history of breast augmentation, reduction, or implants; (3) history of denosumab (a monoclonal antibody that binds RANKL) use in the previous 6 months; (4) history of selective estrogen receptor modulators use in the previous 6 months. We used a modification of the National Comprehensive Cancer Network definition [28], which does not require the measurement of plasma hormone levels to define postmenopausal status. A woman was considered postmenopausal if she had a prior bilateral oophorectomy, was age 60 or older, or if under age 60, had been amenorrheic for at least 12 months.

On the day of the screening mammogram, study participants completed a blood draw and responded to a questionnaire on breast cancer risk factors. Blood samples were processed and stored at − 80 °C within 60 min of collection. A study coordinator measured study participants’ heights using a stadiometer and weights using the OMRON Full Body Sensor Body Composition Monitor and Scale model HBF-514FC. Body mass index (BMI) was derived by dividing current weight (kg) by height (m) squared (kg/m^2^). Approval for the study was granted by the Institutional Review Board at Washington University School of Medicine, St. Louis, MO. All participants provided written informed consent to participate in the study.

### Mammographic breast density assessment

We used Volpara [version 1.5, (Matakina Technology Limited, Wellington, New Zealand)] to obtain automated, objective MBD measurements. Volpara density measurements are highly reproducible [29–31]. Volpara uses a relative physics approach and a computerized algorithm that compares. X-ray attenuation at each pixel to a reference pixel within the breast that is assumed to comprise all adipose/non-dense tissue. Using known X-ray attenuation coefficients for fibroglandular/dense and non-dense tissue, Volpara can then estimate the relative thickness of dense and non-dense tissue at each pixel in the image. As the pixel dimensions are known, these thickness estimates can then be converted to volumes and summed across the breast to determine the absolute volumes of dense volume (DV, cm^3^), and non-dense volume (NDV, cm^3^) in cubic centimeters. i.e., tissue volume at each pixel = tissue thickness × pixel width × pixel length. The total breast volume is determined using Volpara's proprietary segmentation of the breast and model of the breast edge under compression, and the reported compressed breast thickness. The volumetric breast density (%), can then be determined by taking the ratio of the absolute dense volume to the total breast volume, expressed as a percentage. i.e., Volumetric breast density (VPD, %), % = (volume fibroglandular tissue/volume breast) × 100. In comparison with Breast Imaging Reporting and Data System (BI-RADS) fifth edition, Volpara VPD ranges from 0.5 to 34.5%, which translate to: < 3.5% (a, almost entirely fatty breasts); ≥ 3.5–< 7.5% (b, scattered areas of fibroglandular density); ≥ 7.5–< 15.5% (c, heterogeneously dense breasts); ≥ 15.5 (d, extremely dense breasts).

### Plasma gene expression

We performed RNA profiling to quantify gene expression in the plasma, not in the breast tissue to gain further knowledge into how these biomarkers are associated with MBD outside that gleamed from circulating protein levels alone. While mRNA expression and protein levels are correlated across cell lines [32, 33], many factors, including post-translational stability influence circulating protein levels, hence, the correlations of mRNA and their circulating protein levels may be weak [34].

We designed a custom NanoString nCounter codeset for quantitative RNA profiling of the following genes: (1) hormones: prolactin (PRL), progesterone receptor (PGR), estrogen receptor 1 (ESR1), signal transducer and activator of transcription 1 (STAT1), and signal transducer and activator of transcription 5 (STAT5); (2) RANK pathway: RANK, RANKL, tumor necrosis factor receptor superfamily member 13B (TNFRSF13B), tumor necrosis factor receptor superfamily member 18 (TNFRSF18) and osteoprotegerin (OPG). We selected these genes based on data from preclinical studies suggesting crosstalk between the RANK pathway markers (RANK, RANKL, OPG) [35, 36] and specific hormone signaling (PRL, ESR1, PGR, STAT1, and STAT5) [27, 37–39]. TNFRSF13 and TNFRSF18 are also RANK pathway markers that could have biological relevance, but there is limited or no data on their associations with MBD. Thus, we designed targets for those genes as well with NanoString.

RNA profiling for gene expression was performed at the McDonnell Genome Institute, Washington University School of Medicine in St. Louis. Gene expression levels were measured in plasma RNA isolated, using the NanoString “nCounter XT Codeset Gene Expression Assays” protocol (NanoString Technologies, Seattle, WA, USA). Quality control was performed as recommended by the manufacturer. This NanoString protocol has been validated extensively in tissue [40–43] and blood [44, 45].

Plasma samples were processed according to the manufacturer's recommendations. Following hybridization, samples were processed on the NanoString Prep Station where they were purified and immobilized on a sample cartridge for data collection. The output for each sample was imported into nSolver Analysis Software for Quality Control and analysis. Binding densities ranged from 0.09 to 0.34. Digital transcript counts from the NanoString nCounter assay were normalized using the housekeeping genes following the manufacturer’s guidelines.

### Statistical analyses

Statistical analyses were performed with the NanoString nSolver Analysis System 4.0 (NanoString Technologies) using the Advanced Analysis package 2.0 and its custom analysis pipeline. VPD, DV, and NDV were all log-transformed to ensure the normality of the residuals. All analyses were performed on curated log^2^ transformed normalized counts. We evaluated correlations between the genes as well as between the genes and age and BMI using Pearson correlation (*r*). In addition, we evaluated correlations of the genes adjusted for age and BMI. We also performed correlation analysis in a subset of our study participants (82 women with dense breasts) who had both circulating RANK, RANKL, and OPG and mRNA gene expression data. Genes were tested for differential expression to MBD and adjusted for the following confounding variables: race (Non-Hispanic white/African-American/Others), current age (continuous, years), BMI (continuous, kg/m^2^), age at menarche (continuous), menopausal hormone therapy use (ever/never), parity, and age at first birth (continuous). For each gene expression, a single linear regression was fit using all selected variables to predict expression. The fold change is then estimated using a simplified negative binomial model, presented here as ‘linear fold change.’ The 95% confidence interval for the linear fold change is also presented, along with a *p* value. A value of *p* < 0.05 was considered statistically significant. These linear fold changes are herein discussed in terms of percentage increases or decreases such that a linear fold change of 1.04 corresponds to a 4% increase in MBD, and a 0.96 linear fold change corresponds to a 4% decrease in MBD. We further used multinomial logistic regression models to evaluate the associations of growth factor gene expression with categories of VPD, adjusted for confounders.

## Results

The mean age of the study participants was 57.9 years (Table 1). The mean BMI was 31.3 kg/m^2^, which is consistent with the BMI of women attending screening mammograms at the Joanne Knight Breast Health Center. Many participants were Non-Hispanic White (62%) and African-American (35.6%). The mean VPD was 6.2% (BI-RADS category b). The mean DV and NDV were 121.2 cm^3^ and 2134.4 cm^3^, respectively.

**Table 1:** Characteristics of 368 postmenopausal women recruited during annual screening mammogram at the Joanne Knight Breast Health Center, Washington University School of Medicine, St. Louis, MO

Characteristic	Number	Mean ± SD/percentages^a^
Age (years)	368	57.9 ± 3.8
Age at menarche (years)	361	12.8 ± 1.7
Body mass index (kg/m^2^)	367	31.3 ± 7.7
Race/ethnicity		
Non-Hispanic White	228	62.0
Black or African-American	131	35.6
Other	9	2.4
Education		
High school or less than high school	64	17.4
Post high school training or some college	106	28.8
College graduate	106	28.8
Postgraduate	90	24.5
Missing	2	0.5
Alcohol use		
No	149	40.5
Yes	217	59.0
Missing	2	0.5
Family breast cancer history		
No	271	73.6
Yes	91	24.7
Missing	6	1.6
Parity and age at first birth		
Nulliparous	63	17.1
1–2 children, < 25 years	86	23.4
1–2 children, 25–29 years	64	17.4
1–2 children, ≥ 30 years	55	15.0
≥ 3 children, < 25 years	66	17.9
≥ 3 children, ≥ 25 years	33	9.0
Missing	1	0.3
Breast feeding		
No	139	37.8
Yes	164	44.6
Not applicable	64	17.4
Missing	1	0.3
Menopausal hormone therapy use		
No	245	66.6
Yes	122	33.2
Missing	1	0.3
Mammographic breast density		
Volumetric percent density (%)	368	6.2 ± 4.1
VPD < 3.5%	52	
VPD ≥ 3.5% and < 7.5%	234	
VPD ≥ 7.5% and < 15.5%	68	
VPD ≥ 15.5%	14	
Dense Volume (cm^3^)	368	121.2 ± 125.1
Non-dense Volume (cm^3^)	368	2134.4 ± 2062.6

^a^Mean ± Standard deviation (SD) presented for continuous variables. Percentages presented for categorical variable

There were positive correlations between progesterone and OPG plasma gene expression (*r* = 0.65, *p* value < 0.0001), STAT1 and STAT5 (*r* = 0.59, *p* value < 0.0001) plasma gene expression (Table 2), ESR1 and progesterone plasma gene expression (*r* = 0.43, *p* value < 0.0001) as well as prolactin and STAT5 (*r* = 0.43, *p* value < 0.0001) plasma gene expression. TNFRSF18 plasma gene expression was positively correlated with the nine other markers. RANK plasma gene expression was negatively correlated with prolactin, STAT1, and STAT5 plasma gene expression. BMI was weakly inversely correlated with gene expression of RANK pathway markers (Table 2) but not with hormone gene expression, and further adjusting the correlations for age and BMI had negligible impact on the correlation coefficients (Additional file 1: Table S1).

**Table 2:** Correlations between hormones and RANK pathway gene expression in postmenopausal women

Gene	Mean (Range)	Age	BMI	PRL	ESR1	PGR	STAT1	STAT5	RANK	RANKL	OPG	TNFRSF13B	TNFRSF18
PRL	5.81 (4.60–6.83)	0.05	− 0.05	1.00	0.01	**0.25**	**0.25**	**0.39**	− **0.16**	0.02	**0.28**	− **0.18**	**0.24**
		*p* = 0.34	*p* = 0.28		*p* = 0.79	***p* < 0.01**	***p* < 0.01**	***p* < 0.01**	***p* < 0.01**	*p* = 0.70	***p* < 0.01**	***p* < 0.01**	***p* < 0.01**
ESR1	5.38 (4.42–7.42)	0.01	0.07		1.00	**0.43**	− **0.11**	− 0.09	**0.27**	0.08	**0.41**	**0.20**	**0.22**
		*p* = 0.99	*p* = 0.17			***p* < 0.01**	***p* = 0.04**	*p* = 0.08	***p* < 0.01**	*p* = 0.10	***p* < 0.01**	***p* < 0.01**	***p* < 0.01**
PGR	4.4 (3.08–7.00)	0.08	− 0.04			1.00	− **0.15**	− 0.05	**0.25**	0.10	**0.65**	**0.10**	**0.30**
		*p* = 0.20	*p* = 0.42				***p* < 0.01**	*p* = 0.36	***p* < 0.01**	*p* = 0.05	***p* < 0.01**	***p* = 0.04**	***p* < 0.01**
STAT1	14.62 (10.79–16.86)	0.04	− 0.07				1.00	**0.59**	− **0.17**	− 0.02	− **0.15**	− **0.22**	**0.20**
		*p* = 0.43	*p* = 0.18					***p* < 0.01**	***p* < 0.01**	*p* = 0.65	***p* < 0.01**	***p* < 0.01**	***p* < 0.01**
STAT5	11.93 (8.09–12.99)	0.03	0.06					1.00	− **0.21**	− 0.06	− 0.08	− 0.06	**0.35**
		*p* = 0.50	*p* = 0.22						***p* < 0.01**	*p* = 0.24	*p* = 0.11	*p* = 0.25	***p* < 0.01**
RANK	6.33 (5.72–6.93)	− 0.02	− **0.18**						1.00	**0.15**	**0.17**	**0.19**	**0.32**
		*p* = 0.64	***p* < 0.01**							***p* < 0.01**	***p* < 0.01**	***p* < 0.01**	***p* < 0.01**
RANKL	2.93 (2.16–3.70)	0.07	− **0.16**							1.00	**0.17**	− 0.01	**0.14**
		*p* = 0.19	***p* < 0.01**								***p* < 0.01**	*p* = 0.90	***p* < 0.01**
OPG	4.12 (3.57–4.67)	0.04	− **0.10**								1.00	0.07	**0.25**
		*p* = 0.40	***p* = 0.04**									*p* = 0.14	***p* < 0.01**
TNFRSF13B	6.70 (6.09–7.31)	− 0.12	− **0.12**									1.00	**0.23**
		*p* = 0.02	***p* = 0.02**										***p* < 0.01**
TNFRSF18	7.42 (7.04–7.80)	0.01	− **0.13**										1.00
		*p* = 0.92	***p* = 0.01**										

Pearson’s correlation coefficients were calculated. Gene expression levels were log2 transformed

*BMI* Body Mass Index (kg/m^2^)

^*^Statistically significant correlations (*p* < 0.05) are in bold

Table 3 shows the associations between hormone and RANK pathway plasma gene expression and VPD. Of the 10 markers evaluated ESR1, RANK, and TNFRSF18 plasma gene expression were associated with VPD. A one-unit increase in ESR1, RANK, and TNFRSF18 plasma gene expression was associated with 8% (95% CI 0–15%, *p* value = 0.05), 10% (95% CI 0–20%, *p* value = 0.04), and 4% (95% CI 0–9%, *p* value = 0.04) higher VPD, respectively.

**Table 3:** Associations of hormone, RANK pathway gene expression with volumetric percent density

mRNA gene expression	Linear fold change	Lower confidence limit	Upper confidence limit	*p* value
PRL	1.00	0.95	1.06	0.97
ESR1	1.08	1.00	1.15	0.05
PGR	1.07	0.94	1.22	0.29
STAT1	1.00	0.94	1.07	0.96
STAT5	0.98	0.95	1.02	0.29
RANK	1.10	1.00	1.20	0.04
RANKL	1.10	0.94	1.29	0.26
OPG	1.10	0.94	1.28	0.23
TNFRSF13B	1.02	0.95	1.11	0.56
TNFRSF18	1.04	1.00	1.09	0.04

Multivariable model adjusted for race (Non-Hispanic White, Black or African-American, Other), current age (continuous), BMI (continuous), age at first menarche (continuous), menopausal hormone therapy use (Yes, No, Missing), combined parity, and age at first birth (categorical)

We also investigated the associations of plasma gene expression across categories of VPD using multinominal logistic regression models (Table 4). The associations were similar to what we observed evaluating gene expression in the continuous form. We, however, also observed positive associations for RANKL and OPG plasma gene expression when we compared extremes of MBD. Women with extremely dense breasts (VPD > 15.5%; BI-RADS d) had a 73% (95% CI 1.05–2.85, *p* value = 0.03) higher plasma RANKL gene expression, and 86% (95% CI 1.10–3.14, *p* value = 0.02) higher plasma OPG gene expression compared with women with almost entirely fatty breasts (VPD < 3.5%; BI-RADS a). RANKL and OPG plasma gene expression was not higher among women with heterogeneously dense breasts (VPD ≥ 7.5% and < 15.5%; BI-RADS c) compared with women with almost entirely fatty breasts.

**Table 4:** Associations of hormone, RANK pathway gene expression with categories of volumetric percent density

	VPD < 3.5% *N* = 52	VPD ≥ 3.5 and < 7.5% *N* = 234	VPD ≥ 7.5 and < 15.5% *N* = 68	VPD ≥ 15.5% *N* = 14
*Linear fold change (confidence interval), p value*				
PRL	Ref	1.03 (0.93–1.13)	1.08 (0.93–1.24)	0.93 (0.76–1.14)
		*p* = 0.57	*p* = 0.31	*p* = 0.48
ESR1	Ref	0.99 (0.88–1.12)	1.00 (0.85–1.18)	1.28 (0.99–1.64)
		*p* = 0.88	*p* = 1.00	*p* = 0.06
PGR	Ref	1.01 (0.81–1.27)	1.12 (0.84–1.51)	1.41 (0.90–2.23)
		*p* = 0.92	*p* = 0.44	*p* = 0.14
STAT1	Ref	1.06 (0.94–1.19)	1.09 (0.94–1.27)	1.00 (0.79–1.27)
		*p* = 0.36	*p* = 0.27	*p* = 1.00
STAT5	Ref	1.03 (0.96–1.1)	1.01 (0.93–1.10)	0.98 (0.86–1.12)
		*p* = 0.38	*p* = 0.77	*p* = 0.78
RANK	Ref	1.22 (1.05–1.43)	1.23 (1.01–1.51)	1.45 (1.06–1.99)
		*p* = 0.01	*p* = 0.04	*p* = 0.02
RANKL	Ref	0.87 (0.65–1.17)	0.99 (0.68–1.45)	1.73 (1.05–2.85)
		*p* = 0.37	*p* = 0.97	*p* = 0.03
OPG	Ref	1.13 (0.86–1.48)	1.11 (0.78–1.57)	1.86 (1.10–3.14)
		*p* = 0.37	*p* = 0.57	*p* = 0.02
TNFRSF13B	Ref	0.98 (0.85–1.12)	0.89 (0.74–1.07)	1.31 (0.99–1.74)
		*p* = 0.75	*p* = 0.21	*p* = 0.06
TNFRSF18	Ref	1.13 (1.06–1.22)	1.11(1.02–1.22)	1.26 (1.09–1.45)
		*p* = 0.001	*p* = 0.02	*p* = 0.002

*VPD* volumetric percent density

In a subset of our study participants (82 women with dense breasts) who had both circulating RANK, RANKL, and OPG and mRNA gene expression data (Additional file 1: Table S2), we observed mild positive correlations between circulating protein levels and the mRNA gene expression for RANK (*r* = 0.26, *p* value = 0.03), RANKL (*r* = 0.23, *p* value = 0.04) but not for OPG (*r* = − 0.03, *p* value = 0.81).

We further evaluated the associations of plasma gene expression with NDV and DV (Additional file 1: Table S3). A one-unit increase in plasma OPG, PGR, and TNFRSF13B gene expression was associated with 12% (95% CI 4–19%, *p* value = 0.003), 7% (95% CI 0–13%, *p* value = 0.04), and 5% lower (95% CI 1–9%, *p* value = 0.02) NDV, respectively. Only plasma OPG gene expression was associated with DV: a one-unit increase in OPG was associated with 8% (95% CI 0–15%, *p* value = 0.05) lower DV.

## Discussion

To the best of our knowledge, this is the first study to investigate the associations of plasma hormone and RANK pathway gene expression with volumetric measures of MBD in postmenopausal women. We observed positive associations of ESR1, RANK, and TNFRSF18 plasma gene expression with VPD and inverse associations of PGR and OPG plasma gene expression with NDV.

VPD represents the stromal and epithelial components of fibroglandular breast tissue and is positively associated with breast cancer risk [46–48] while NDV represents the adipose component of breast tissue and is inversely associated with breast cancer risk in many studies [46, 49, 50]. Our finding of a positive association of plasma ESR1 gene expression with VPD is similar to that reported for circulating estradiol and percent density in some studies [11, 51, 52], while other studies have reported inverse [12, 16, 17], or no associations between circulating estradiol and percent density [13, 15, 53]. These results are difficult to directly compare as they examine plasma gene expression or circulating hormone levels. Taken together, they suggest that a singular circulating estradiol level, as determined in these studies, may not be sufficient to serve as a reliable proxy for estrogen activity.

ESR1 (ERα) regulates estrogen activity, and the ESR1 gene encodes a transcription factor with an estrogen binding domain, activating domain, and estrogen response element [54, 55]. Once estrogen binds to ESR1, proliferation is induced in both normal and neoplastic breast epithelial cells through ESR1 signaling of estrogen-responsive genes [56]. Thus, if ESR1 gene expression is increased this may lead to greater proliferation of breast tissue, culminating in greater MBD and increased breast cancer risk. This mechanism could explain the association between plasma ESR1 gene expression and VPD identified in our study.

Interestingly, a previous study using data from 79 women reported a positive association of serum estradiol level but reported an inverse association of breast tissue ESR1 gene expression with percent density in postmenopausal women [21]. The authors of this study pointed out that increased levels of estradiol have also been shown to decrease levels of ESR1 in breast cancer [57] and thus reasoned that the association between reduced ESR1 and high MBD may reflect high levels of plasma estradiol. Therefore, longitudinal studies that concomitantly explore the associations between circulating estradiol and plasma ESR1 gene expression with MBD are warranted in postmenopausal women.

The associations of serum RANK and RANKL gene expression we observed are similar to our results in premenopausal women [22]. RANK causes mammary epithelial cell proliferation perhaps via upregulation of cyclin D1 [58], and RANKL is essential for the development, formation, and differentiation of mammary glands [25, 59]. Some studies have reported associations of the RANK pathway with breast cancer pathogenesis, while others have not [60–63]. The positive association of plasma RANKL gene expression with MBD was limited to when we compared women at the extremes of MBD profiles, which suggests a nonlinear association between RANKL gene expression and MBD.

In addition, women with extremely dense breasts had higher OPG gene expression than women with almost entirely fatty breasts. OPG gene expression was inversely associated with DV and NDV. The findings were unexpected given that OPG competes with RANK for RANKL binding, thereby blocking RANK activation [64]. Hence, we hypothesized that OPG mRNA expression would be negatively associated with VPD and DV. Our findings are similar to a study that reported low OPG serum levels to be associated with high mammographic breast density (VPD and DV) [65] and contrary to another study that found no association between OPG and VPD [63]. Furthermore, some findings from preclinical studies show that OPG expression in tissue may be associated with breast tumor formation [36, 66]. Thus, the association of OPG with mammographic breast density remains unclear and deserves to be studied further. Due to the limited data on the role of OPG in breast proliferation, development, and function in humans, clinical studies are needed to elucidate the role of OPG in MBD and breast cancer development as well as how these associations may be mediated by estrogen and progesterone, given the correlations we observed across these genes.

Plasma progesterone receptor gene expression was not associated with VPD but was associated with NDV. Some studies have reported associations between circulating progesterone and percent breast density and percent dense area [13, 67, 68], while others have not [12, 15, 17]. Our finding is similar to another study that found a positive association of progesterone with absolute non-dense breast volume in premenopausal women [69]. NDV is inversely associated with breast cancer risk [46, 49, 50], suggesting that elevated progesterone gene expression may be associated with elevated breast cancer risk in postmenopausal women.

We found no association between plasma prolactin gene expression and VPD, consistent with findings from previous studies on circulating prolactin and percent dense area in postmenopausal women [17, 70], but not with others that have reported positive associations [12, 71]. One study used an immunoassay rather than circulating hormones to quantify prolactin levels and determined that postmenopausal women with high prolactin immunoassay profiles had higher breast dense area and lower non-dense area than those with lower prolactin immunoassay readings [72].

We observed correlations between plasma RANK pathway gene expression and hormone gene expression, which is an indication of the crosstalk between these markers and may provide further biological insights into the complex pathways through which the markers influence MBD and breast cancer risk. Progesterone upregulates RANKL expression [24, 25] and interacts with the RANK/RANKL pathway through signal transducer and activator of transcription (STAT) signaling [26, 27], while OPG mRNA transcription in healthy breast tissue is regulated by estrogen [73, 74]. Further clinical studies are needed to characterize the interrelationships between these markers and how they influence MBD and breast cancer development. The inverse correlations of BMI with OPG gene expression we observed are similar to what has been reported for their circulating levels [75] but we did not observe positive correlations of BMI with ESR1 and PRL gene expression, in contrast to what has been reported for their circulating levels [76]. Other studies evaluating correlations of BMI with hormone gene expression are needed.

Our study has several strengths. Study participants were recruited among women attending annual routine screening mammograms. We analyzed plasma hormone gene expression rather than circulating hormone levels, and we did compare the gene expression to protein levels in a subset of participants. However, we did not compare the mRNA gene expression in the plasma to the gene expression in the breast tissue since study participants were cancer-free women recruited during their annual screening mammogram. Future studies integrating plasma gene expression, target tissue gene expression, and circulating protein levels as biomarkers in elucidating MBD for breast cancer risk are encouraged.

One limitation of our study is that the sample size was not large enough to perform mediation analyses between hormone and RANK gene expression on MBD or to conduct analyses stratified by BMI and race. We did not profile plasma gene expression of other hormones such as androgens and sex hormone-binding globulin, and parathyroid hormone, a known regulator of the RANK pathway. Future studies evaluating how these hormones are associated with MBD will be needed.

## Conclusions

In conclusion, we observed positive associations of ESR1, RANK, and TNFRSF18 plasma gene expression with VPD in postmenopausal women. Women with extremely dense breasts had higher RANKL and OPG plasma gene expression than women with entirely fatty breasts. These findings require validation within other study populations.

## Supplementary Information

**Additional file 1**. ** Table S1:** Correlations between Hormones and RANK Pathway Gene Expression in Postmenopausal Women, adjusted for age and BMI. **Table S2:** Correlations between Circulating RANK, sRANKL, OPG Protein and Their Gene Expression Levels Among 82 Women with Dense Breasts. **Table S3:** Associations of Hormone, RANK Pathway Gene Expression with Non-Dense Volume and Dense Volume.

## Data Availability

All data generated or analyzed during this study are included in this published article and its Additional file 1.

## Abbreviations

MBD: Mammographic breast density
RANK: Receptor activator of nuclear factor-kB
RANKL: Receptor activator of nuclear factor kappa-Β ligand
VPD: Volumetric percent density
DV: Dense volume
NDV: Non-dense volume
PRL: Prolactin
PGR: Progesterone receptor
ESR1: Estrogen receptor 1
STAT1: Signal transducer and activator of transcription 1
STAT5: Signal transducer and activator of transcription 5
TNFRSF13B: Tumor necrosis factor receptor superfamily member 13B
TNFRSF18: Tumor necrosis factor receptor superfamily member 18
OPG: Osteoprotegerin
