# Supplementary material for: Hormone and receptor activator of NF-κB (RANK) pathway gene expression in plasma and mammographic breast density in postmenopausal women
Source: Breast Cancer Res. 2022 Apr 14;24:28. doi: 10.1186/s13058-022-01522-2 (PMC9008951; doi:10.1186/s13058-022-01522-2)
Supplement: Supplementary file 1 — Additional file 1. Table S1: Correlations between Hormones and RANK Pathway Gene Expression in Postmenopausal Women, adjusted for age and BMI. Table S2: Correlations between Circulating RANK, sRANKL, OPG Protein and Their Gene Expression Levels Among 82 Women with Dense Breasts. Table S3: Associations of Hormone, RANK Pathway Gene Expression with Non-Dense Volume and Dense Volume. [file 13058_2022_1522_MOESM1_ESM.docx]

| **Additional file 1: Table S1: Correlations between Hormones and RANK Pathway Gene Expression in Postmenopausal Women, adjusted for age and BMI**^a^ | | | | | | | | | | |
| --- | --- | --- | --- | --- | --- | --- | --- | --- | --- | --- |
| Gene | PRL | ESR1 | PGR | STAT1 | STAT5 | RANK | RANKL | OPG | TNFRSF13B | TNFRSF18 |
| **PRL** | 1.00 | 0.01  *p*=0.72 | **0.25**  ***p<*0.01** | **0.25**  ***p*<0.01** | **0.39**  ***p<*0.01** | **-0.15**  ***p<*0.01** | 0.02  *p*=0.59 | **0.28**  ***p<*0.01** | **-0.18**  ***p<*0.01** | **0.24**  ***p<*0.01** |
| **ESR1** |  | 1.00 | **0.43**  ***p<*0.01** | **-0.11**  ***p<*0.01** | **-0.09**  ***p=*0.01** | **0.27**  ***p*<0.01** | **0.08**  ***p=*0.02** | **0.41**  ***p<*0.01** | **0.20**  ***p<*0.01** | **0.22**  ***p<*0.01** |
| **PGR** |  |  | 1.00 | **-0.15**  ***p*<0.01** | -0.05  *p*=0.19 | **0.25**  ***p*<0.01** | **0.10**  ***p*<0.01** | **0.65**  ***p<*0.01** | **0.10**  ***p<*0.01** | **0.30**  ***p<*0.01** |
| **STAT1** |  |  |  | 1.00 | **0.59**  ***p*<0.01** | **-0.16**  ***p*<0.01** | **-0.02**  *p*=0.50 | **-0.14**  ***p<*0.01** | **-0.22**  ***p<*0.01** | **0.20**  ***p<*0.01** |
| **STAT5** |  |  |  |  | 1.00 | **-0.21**  ***p*<0.01** | **-0.06**  *p=*0.10 | **-0.08**  ***p=*0.02** | -0.06  *p=*0.11 | **0.35**  ***p<*0.01** |
| **RANK** |  |  |  |  |  | 1.00 | **0.15**  ***p*<0.01** | **0.17**  ***p<*0.01** | **0.19**  ***p<*0.01** | **0.31**  ***p<*0.01** |
| **RANKL** |  |  |  |  |  |  | 1.00 | 0.02  *p=*0.64 | -0.01  *p=*0.82 | **0.14**  ***p<*0.01** |
| **OPG** |  |  |  |  |  |  |  | 1.00 | **0.07**  ***p=*0.04** | **0.25**  ***p<*0.01** |
| **TNFRSF13B** |  |  |  |  |  |  |  |  | 1.00 | **0.23**  ***p<*0.01** |
| **TNFRSF18** |  |  |  |  |  |  |  |  |  | 1.00 |
| ^a^Pearson’s partial correlation coefficients were calculated adjusting for age (continuous) and BMI (continuous). Gene expression levels were log2 transformed.  *Statistically significant correlations (p<0.05) are in bold. | | | | | | | | | | |

**Additional file 1: Table S2: Correlations between Circulating**

**RANK, sRANKL, OPG Protein and Their Gene Expression**

**Levels Among 82 Women with Dense Breasts***

| **Circulating Protein and Gene Expression** | **Correlation^a^** |  |
| --- | --- | --- |
| RANK | 0.26 (*p*=0.03) |  |
| sRANKL | 0.23 (*p*=0.04) |  |
| OPG | -0.03 (*p*=0.81) |  |
| ^*^Dense breast was defined as volumetric percent density >= 7.5%  ^a^ Pearson correlation coefficients were derived using the original gene  expression data | | |

**Additional file 1: Table S3: Associations of Hormone, RANK Pathway Gene Expression with**

**Non-Dense Volume and Dense Volume**

| Non-Dense Volume | | | | |
| --- | --- | --- | --- | --- |
| mRNA gene expression | Linear fold change | Lower confidence limit | Upper confidence limit | P-value |
| **PRL** | 1.02 | 0.99 | 1.05 | 0.26 |
| **ESR1** | 0.97 | 0.93 | 1.00 | 0.08 |
| **PGR** | 0.93 | 0.87 | 1.00 | 0.04 |
| **STAT1** | 1.02 | 0.99 | 1.05 | 0.26 |
| **STAT5** | 1.02 | 1.00 | 1.04 | 0.14 |
| **RANK** | 0.96 | 0.91 | 1.00 | 0.08 |
| **RANKL** | 0.94 | 0.86 | 1.03 | 0.20 |
| **OPG** | 0.88 | 0.81 | 0.96 | 0.003 |
| **TNFRSF13B** | 0.95 | 0.91 | 0.99 | 0.02 |
| **TNFRSF18** | 0.98 | 0.96 | 1.00 | 0.09 |
| Dense Volume | | | | |
| mRNA gene expression | Linear fold Change | Lower confidence limit | Upper confidence limit | *p*-value |
| **PRL** | 1.01 | 0.98 | 1.04 | 0.50 |
| **ESR1** | 0.99 | 0.95 | 1.03 | 0.58 |
| **PGR** | 0.95 | 0.89 | 1.02 | 0.17 |
| **STAT1** | 1.01 | 0.98 | 1.05 | 0.43 |
| **STAT5** | 1.01 | 0.99 | 1.03 | 0.38 |
| **RANK** | 0.98 | 0.94 | 1.03 | 0.48 |
| **RANKL** | 0.97 | 0.89 | 1.06 | 0.50 |
| **OPG** | 0.92 | 0.85 | 1.00 | 0.05 |
| **TNFRSF13B** | 0.97 | 0.93 | 1.01 | 0.15 |
| **TNFRSF18** | 0.99 | 0.97 | 1.02 | 0.58 |

Multivariable model adjusted for race (Non-Hispanic White, Black or African American, Other), current age (continuous), BMI (continuous), age at first menarche (continuous), menopausal hormone therapy use (Yes, No, Missing), parity and age at first birth (categorical).
